# Ovarian cancer in Lebanese women: a 12-year comparative epidemiological study and trend analysis

**DOI:** 10.3332/ecancer.2022.1409

**Published:** 2022-06-13

**Authors:** Antonio El Kareh, Steven Safi, Théa Kaady, Said El Hage, Elie Mokled, Elise Assouad, Elie Snaifer, Reine Nader

**Affiliations:** 1 Faculty of Medical Sciences, Lebanese University, Hadath, Lebanon; 2 School of Medicine, Lebanese American University, Byblos, Lebanon; 3 Middle East Institution of Health University Hospital, Bsalim, Lebanon; 4 Saint Georges Hospital University Medical Center, Beirut, Lebanon; 5 Snaifer Clinic, Cedim Center Abraj, Furn el Chebbak, Lebanon; † Co-first authors.

**Keywords:** epidemiology, gynaecology, Lebanon, oncology, ovarian cancer

## Abstract

**Purpose:**

Over the last decade, Lebanon has experienced an increase in the rate of cancer patients. The aim of this study is to investigate the incidence rates of ovarian cancer in Lebanese women over a period of 12 years and to compare them to other countries.

**Methods:**

Data were collected from the Lebanese National Cancer Registry for the time period 2005–2016 (inclusive). Data from other countries were retrieved from an online database ‘Cancer Incidence in Five Continents’. The age-specific and age-standardised incidence rates (ASIR) were calculated and analysed using Joinpoint regression.

**Results:**

Ovarian cancer ranked seventh among the commonest cancers in Lebanese women in the studied time frame. Approximately 189 new cases were reported every year, with an average age-standardised incidence rate of 7.88 (per 100,000 women). Ovarian cancer showed a significantly decreasing trend in the 12 years of study. Lebanon had one of the highest ASIR for ovarian cancer among regional countries and randomly selected countries.

**Conclusion:**

Lebanon presented a high ASIR for ovarian cancer compared to regional countries, and was placed among the top ASIRs compared to countries worldwide. However, with the decreasing ovarian cancer trends, it is important to implement efficacious awareness in order to detect all OC cases.

## Introduction

Cancer is the second most common cause of death worldwide, only surpassed by diseases related to the cardiovascular system [1]. One specific type, ovarian cancer (OC), is a highly lethal and relatively common type of gynaecological cancer [2]. It is the eighth most common cancer type in women, and the eighth commonest cause of cancer death among females in the world. In fact, 313,959 new cases of OC were reported in 2020 globally, accounting for 3.4% of all new female cancer cases, and deaths due to OC represented around 4.7% of all cancer-related deaths [3]. Ovarian neoplasms are classified by the World Health Organisation according to the probable tissue of origin. These categories are surface epithelial (65%), germ cell (15%), sex cord-stromal (10%), metastases (5%) and miscellaneous (5%) [4].

Numerous risk factors are associated with OC. Some predisposing factors are classified as demographic (age and geographic location), reproductive (menstrual-related factors, age of menarche and menopause), gynaecologic diseases, hormonal, genetic (family history and Breast Cancer Gene 1 mutations) and lifestyle-related [5]. At a regional level, OC is a relatively common disease in the 22 Arab countries, with one of the highest incidences in the world. It is most commonly reported with breast cancer but is still considered underreported and underdiagnosed [6].

At a national level, the National Cancer Registry (NCR) established by the Ministry of Public Health (MoPH) is a database that collects cancer reports from all around the country [7].

The sole purpose of this study is to analyse the epidemiological aspects of OC among Lebanese women between 2005 and 2016, in order to display its trend, all while comparing the national rates of the Lebanese population at both regional and international levels.

## Materials and methods

### Data collection and processing

The number of incident OC cases was collected from the Lebanese NCR for the time period 2005–2016. The NCR is an information system established in order to collect, store, manage and analyse data on cancers throughout Lebanon. The main objectives of this registry are to measure cancer incidence; describe cancer by time, place and disease; and finally provide a national database for further epidemiological research [8]. The NCR tables are available for the public on the Lebanese MoPH official website (https://www.moph.gov.lb/en). The OC incident cases were grouped according to age. Population size data were gathered from the latest World Population Prospect [9]. The age-specific incidence rates, which are the number of new cases for a specific age group during a certain period, were calculated by dividing the number of cases for a certain age group by the corresponding population size and are demonstrated as the number of cases per 100,000 persons. Since it is important to standardise the incidence rates for comparing the burdens of diseases between countries, the age incidence rates were standardised and expressed as the age-standardised incidence rates (ASIR). The latter represent the incidence rates that would have been found if the population considered in the study had the same age composition as a reference population. In this study, the incidence rates were standardised according to the World Standard Population [10]. The age-standardised incidence rates were corrected for cases of unknown age groups, and the correction procedure involved multiplying the age-standardised incidence rate based on cases of known age group with *T/K*, where *T* is the total number of cancer cases and *K* is the number of cancer cases of known age.

### Data analysis

The Joinpoint regression software, a software provided by the National Cancer Institute and commonly used in analysing cancer trends, was used to analyse the age-specific rates and the age-standardised incidence rates, with a significance level of 0.05. The regression was used to describe the trends of OC in Lebanon among the female population, and this by studying the annual percent changes (APC) in incidence rates and age-standardised incidence rates between 2005 and 2016.

The calculated age-standardised incidence rates were compared to those of regional countries (located in the Asian continent) and other selected countries, which were chosen using a website that originates random names of countries through the simple randomisation method. The International Agency for Research on Cancer and the International Association of Cancer Registries publish, every 5 years, high-quality comparable statistics on the incidence of cancers in many countries worldwide, which can be found on the Cancer Incidence in Five Continents volume XI (CI5XI) [11], and, from this online database, the standardised rates and age-specific rates of the countries were collected.

In addition, we used the calculated incidence rates available from 2005 to 2016 to estimate the incidence rates of OC in Lebanon for the time period 2017–2030. The incidence rates for this 12-year period, considered as the dependent variable, were fitted according to the years, the independent variable, into a logarithmic model using Microsoft Excel. This model was used to predict the incidence rates for the 14-year period. Linear and log-linear regression models have been found to be the most practical methods for the estimation of future patterns of cancer incidence. Unlike models based on assumptions of a normal distribution, the log-linear model assumes a Poisson distribution of the number of cases, resulting in a more reliable prediction [12]. Ovarian cancer incidence rates were estimated throughout the upcoming years using the logarithmic model.

An Institutional Review Board approval was unnecessary since all data in this descriptive epidemiological study were obtained from publicly available databases.

## Results

A total of 2,268 cases of OC were reported in Lebanon between 2005 and 2016, accounting for 1.79% of the total cancer cases in the 12-year span, considering that the total incidence of female cancers was of 64,536 in that period. For that, OC ranked seventh among the most prevalent cancers in Lebanese women. Breast cancer ranked first with a total of 24,080 reported cases in women from 2005 to 2016, followed by respiratory tract cancer and colon cancer with 3,636 and 3,608 cases, respectively, as shown in Table 1, which displays the ranking of cancers among females.

An average of 189 cases were reported every year, most of them being patients above 40 years of age (89.15%). The ASIR averaged 7.88 per 100,000 women, fluctuating between 7.24 and 9.86 per 100,000 women throughout the 12-year period (Table 2).

**Figure 1.**
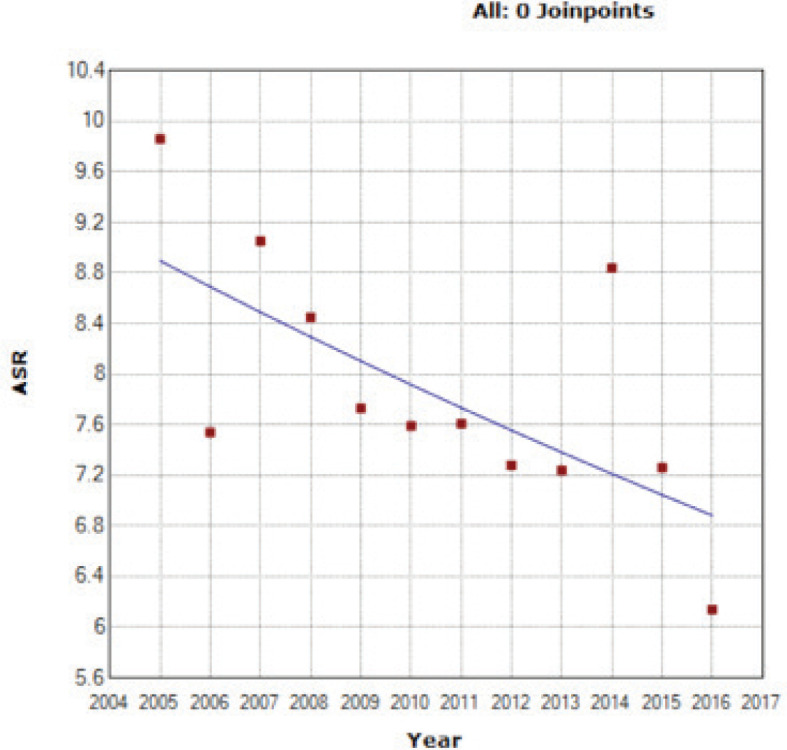
ASIR for OC (per 100,000 females) between 2005 and 2016.

An APC of the ASIR of −2.30* was provided through the Joinpoint software, showing a significant decreasing trend of the OC rates in the 12 years of study (Figure 1) (‘*’ indicates that the APC is significantly different from 0 at the *α* = 0.05 level).

**Figure 2.**
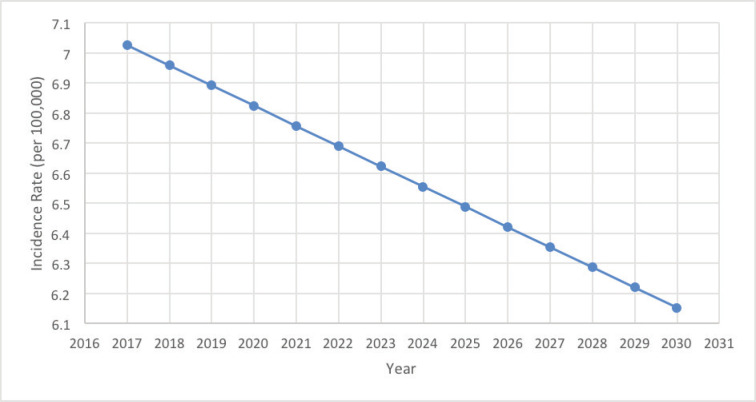
14-year OC incidence rates projection in Lebanon.

Using the Joinpoint regression on the age-specific rates between 2005 and 2016 to obtain the APCs of each age group (Table 2), two of the latter recorded remarkable changes. In fact, the 25–29 years age group showed a significant decrease in its ASIRs with an APC of −9.38* (*p* = 0.038; CI [−17.33 ; −0.67]), while the 50–54 years age group showed a significant increase in its ASIRs with an APC of +3.63* (*p* = 0.038; CI [0.23;7.14]). Moreover, the incidence rates of OC proved to be increasing with age, and are most prominent in the 50–54 years age group, with a total of 320 ovarian cancer cases throughout the 12-year period. In addition, rates are expected to decrease gradually between 2017 and 2030, reaching an incidence rate as low as 6.15 per 100,000 females (Figure 2).

Finally, age-specific rates and age-standardised incidence rates of OC at the regional level and randomly selected countries are presented in Table 3.

## Discussion

As depicted in the results section, ovarian cancer stands as the seventh most common type of cancer in the Lebanese female population between 2005 and 2016 (Table 1), contributing to 1.79% of the total cancer cases in the studied time frame. This emphasised incidence could be due to genomic modifications and mutations among the Arab population, specifically the Lebanese population. In fact, the BRCA1/2 mutations among Arab patients with OC proved to be one of the main pillars to its prevalence. Most of the studies we found focused on germline BRCA 1/2 mutations, mainly due to the fact that these genes present the highest risk of breast and ovarian cancer. These mutations also correlate with the relatively increased ASIR among some of the regional Arab countries near Lebanon, such as Qatar and Bahrain, recording ASIRs of 8 and 6.9, respectively (Table 3).

On the other hand, the use of oral contraceptives was found to decrease the risk of ovarian cancer in the general population [13], as well as in BRCA1/2 mutation carriers [13, 14]. According to El Khoury *et al* [15], firstly, the contraceptive prevalence rate (CPR) in Lebanon was found to equal 55.6% in 2017, with periodic abstinence being the most used method of contraception (50.9%) and oral contraception representing only 32.7% of these contraceptive methods, which could be the explanation of the enhanced incidence of OC in Lebanon in comparison with other countries. Indeed, Qatar, which is characterised by an ASIR of 8, has also a low CPR (47.8%), with oral contraceptives representing only 30.1% of the methods of contraception used [16], but in Costa Rica, the CPR with any contraceptive method and the CPR with modern contraceptive methods (including oral contraceptives) were of 75.2% and 73.9%, respectively, leading to the relatively low ASIR (5.3) [17].

Nonetheless, the use of oral contraceptives as prevention for ovarian cancer is not permitted, because it is known to increase the risk of breast cancer in the general population but not in the BRCA1/2 mutation carriers, although the latter correlation cannot be excluded [13], as well as the risk of cervical cancer [18]. Yet, in comparison with never use, ever use of oral contraceptives lead to a significant decrease in colorectal and endometrial cancers [18], and since colon cancer is the third most prevalent cancer in Lebanese women (Table 1), the use of oral contraceptives as prevention in patients at risk for both ovarian and colon cancers could be a possibility depending on each case; thus, more studies are yet to be conducted regarding that matter.

Meanwhile, the 25–29 years age group presents the highest childbirth rate (109.91 births per 1,000 women) [19], and since it has been stated that ovarian mass [20], and mostly cancer [21], is extremely rare during pregnancy, it is not surprising to obtain a drop in the ovarian cancer ASIRs in this specific age group.

In addition to that parity was proven to be associated with a reduced ovarian cancer risk on one hand [22, 23], and, on the other, the fertility rates of Lebanese women were found to decrease from 2.080 in 2005 to 1.900 in 2008, and then increase to 2.086 in 2016 [24]. So, this slight overall increase in the fertility rates could be responsible for a part of the reduced ovarian cancer risk in Lebanon.

In addition, epithelial ovarian cancer is an age-related disease, and is thought to be mainly post-menopausal [25, 26]. This can be extensively seen in the Lebanese female population, as proved in Table 2, in which an increased incidence of this cancer was more pronounced in women over 64 years of age, with an ASIR of 33.70, placed first among the age groups. This correlation is yet to be explained, but researchers have claimed that the earlier the diagnosis, the better the prognosis [27, 28].

Moreover, the Lebanese population itself can be considered a rather obese one, seeing the fact that most Lebanese adults, 20 years of age and older, are overweight (53.0%, body mass index (BMI) ≥ 25 kg/m^2^), and 17% are obese (BMI ≥ 30 kg/m^2^) [29]. In fact, various studies have proved that an advanced correlation exists between obesity/elevated body mass index (BMI) and the onset of ovarian cancer [30]. In a study conducted by Rodriguez *et al* [31], a 36% increase in the risk of ovarian cancer among obese people who have never used post-menopausal oestrogen treatment was found, leading to the conclusion that obesity can increase the mortality of ovarian cancer [32]. That explains the fact that among its regional neighbours in the Middle Eastern Region, Lebanon ranked second with an ASIR of 7.9 per 100,000 females, followed by Qatar which ranked first with an ASIR of 8 per 100,000 females (Table 3).

Furthermore, throughout the years, the ASIR for OC among females in Lebanon has been subject to a downfall, and the projection towards year 2030 takes a solid dip (Figures 1 and 2). This decrease in ovarian cancer incidence was also seen in Austria (APC of −3.7 from 2000 to 2009) and in New Zealand (APC of −1.7 from 2004 to 2013), while an increase was witnessed in the Republic of Korea (APC of +2.1 from 2005 to 2014) [33] and in the regional country Saudi Arabia (fourfold increase) [34].

This unexplained reduction in the number of diagnosed OC cases could be due to the immense increase in population, with the increased influx of refugees, specifically in 2014 and 2015. In fact, in Lebanon, in 2015, the refugee population represented 30% of the total population. International health assistance has been provided to refugee populations in Lebanon. However, the current humanitarian system has also contributed to the increase in fragmentation of the Lebanese health system [35]. Lebanon has a fragmented and uncoordinated healthcare system, which is highly privatised and based on user fees [36]. However, infectious disease tracing and diagnosis among the Lebanese population, as well as refugees, has been developed within the country through centres, predominately run by non-government organisations (NGOs) through contractual agreements between the Ministry of Public Health and the NGO [37], but this is not similar for non-communicable diseases and cancerous pathologies. This could have led to a total increase in population count, with an unhindered OC case report number, which eventually lead to a consequent downfall in the ASIR throughout the years, explaining the unusual results.

Lastly, in contradiction with our results, breast cancer incidence in Lebanon showed an increase from 2005 to 2015 with an APC of +4.6 [1]. This emphasises the fact that Lebanese women, and the general population, have to be the subjects of extensive awareness campaigns given that early symptoms of ovarian cancer can be detected [38] and that the rise in breast cancer incidence is attributed to the ‘increased attention regarding awareness campaigns’ [1].

## Study limitations

Various limitations were encountered throughout the study. In fact, the Lebanese NCR provided by the Lebanese Ministry of Public Health is the only database the data were collected from. The results are thus dependent on the reliability of the source and the inclusion of refugees. In addition, the Lebanese NCR provides no data on cancer-related mortality rates, limiting the purpose of the study in studying the incidence rates and trends. These limitations halted the full validation of the hypothesis that relates the increase in the incidence of ovarian cancer to the known risk factors.

## Conclusion

Ovarian cancer data provided by the MOPH were collected and ASIRs were calculated for the female gender according to age groups and per year. Lebanon ranked second among the regional countries in terms of ASIR and is placed among the top ASIRs worldwide. The collected data and analysis can help in developing further knowledge on the incidence of ovarian cancer in Lebanon and how to possibly diminish its prevalence and limit the number of cases foreseen in the upcoming years by further limiting and containing the various risk factors. Moreover, with decreasing ovarian cancer trends, it is important to increase the attention of both Lebanese women and the Lebanese healthcare system to the signs and symptoms of ovarian cancer, so the latter’s detection and correct documentation can be possible.

## Conflicts of interest

The authors declare that they have no known competing financial interests or personal relationships that could have appeared to influence the work reported in this paper.

## Funding

This research received no specific grant from any funding agency in the public, commercial, or not-for-profit sectors.

## Authors’ contributions

Antonio El Kareh and Steven Safi: Data processing, analysis and discussion writing

Théa Kaady: Data collection and writing

Elise Assouad: Data collection

Said El Hage: Data collection, assembling and results writing

Elie Mokled: Statistical analysis

Elie Snaifer and Reine Nader: Paper review and correction

## Figures and Tables

**Table 1.** Ranking of OC along with the most common cancers in females in Lebanon between 2005 and 2016.

Rank	Type	Number of cases
1	Breast	24,080
2	Trachea bronchus and lung	3,636
3	Colon	3,608
4	Other skin	3,374
5	Non-Hodgkin lymphoma	3,174
6	Thyroid	2,562
**7**	**Ovary**	**2,267**
8	Corpus uteri	2,171
9	Bladder	1,723
10	Stomach	1,442

**Table 2.** Age-adjusted rates and age-standardised incidence rates for OC cases according to age groups per year, with APC and confidence intervals (CI), in Lebanon between 2005 and 2016.

Year	Age Group																	ASR
	All	0-4y	5-9y	10-14y	15-19y	20-24y	25-29y	30-34y	35-39y	40-44y	45-49y	50-54y	55-59y	60-64y	65-69y	70-74y	75+y	
2005	7.89	0.00	0.00	0.38	0.42	2.28	2.03	1.13	3.75	9.70	17.82	17.12	34.97	55.54	29.20	34.46	45.59	9.86
2006	6.55	0.00	0.00	0.38	0.82	1.35	2.50	1.11	2.43	10.01	12.08	15.87	34.57	19.94	24.56	24.84	41.68	7.54
2007	8.00	0.00	0.00	0.39	0.41	2.24	3.96	2.75	3.60	19.12	11.04	20.74	27.14	34.05	38.13	30.45	38.34	9.05
2008	7.23	0.00	0.46	0.40	0.40	0.00	1.47	3.27	3.58	7.72	23.48	24.07	18.64	40.46	38.50	28.20	24.74	8.45
2009	6.89	0.00	0.00	0.00	0.00	0.88	0.48	4.27	2.94	7.34	14.79	16.50	22.60	33.71	39.76	34.09	24.34	7.73
2010	6.77	0.00	0.00	0.00	1.92	0.42	0.93	1.03	3.99	8.20	14.74	29.52	16.49	30.26	43.24	31.89	17.73	7.59
2011	7.17	0.00	0.00	0.40	0.37	1.60	1.75	0.97	2.17	4.78	16.69	26.62	30.82	19.42	32.81	32.59	37.06	7.61
2012	6.94	0.00	0.43	0.39	1.43	1.49	0.82	2.27	5.60	6.75	18.66	23.37	14.46	12.62	29.83	43.74	34.09	7.28
2013	6.79	0.00	0.00	0.00	0.34	0.00	1.15	2.11	4.76	6.37	17.63	19.39	31.66	20.47	34.87	19.10	27.96	7.24
2014	8.27	0.36	0.38	0.71	1.34	1.00	1.08	2.38	2.25	9.57	13.99	27.91	23.17	32.83	47.38	29.74	39.30	8.84
2015	6.90	0.34	0.00	0.34	1.96	0.64	1.04	3.78	3.00	9.18	13.23	26.25	27.64	24.90	26.73	22.73	22.97	7.26
2016	5.81	0.00	0.00	0.00	0.65	1.58	0.67	1.10	1.24	3.73	11.57	24.02	19.51	16.27	19.35	27.51	16.04	6.14
Ci		-	-	-	-	-	[-17.33; -0.67]	[-8.36; 13.46]	[-10.63; 3.74]	[-12.11; 0.39]	[-5.16; 3.33]	[0.23; 7.14]	[-7.79; 2.83]	[-12.47; 0.34]	[-6.10; 3.64]	[-5.51; 2.47]	[-10.16; 0.70]	[-4.08; -0.50]
APC		-	-	-	-	-	-9.38*	1.97	-3.71	-6.06	-1	3.63*	-2.62	-6.28	-1.35	-1.6	-4.88	-2.30*
*p*-value		-	-	-	-	-	0.038	0.693	0.285	0.062	0.611	0.038	0.303	0.06	0.554	0.395	0.079	0.018
**Final**	**7.10**	**0.06**	**0.11**	**0.28**	**0.84**	**1.12**	**1.49**	**2.18**	**3.27**	**8.54**	**15.47**	**22.61**	**25.14**	**28.37**	**33.70**	**29.95**	**30.82**	**7.88**

**Table 3.** Age-specific rates and age-standardised incidence rates of OC at the regional level and in randomly selected countries.

Gender	Country		Years	Age groups																		ASR(w)
				0-4y	5-9y	10-14y	15-19y	20-24y	25-29y	30-34y	35-39y	40-44y	45-49y	50-54y	55-59y	60-64y	65-69y	70-74y	75-79y	80-84y	85+y	
Female	Regional	China	2008-2012	-	-	-	-	1.7	4.4	2.6	4.5	12.2	14.7	24.5	22.1	15.6	21.1	29.0	29.3	16.8	4.1	**6.9**
		Bahrain	2008-2012	-	-	-	1.4	3.0	-	1.9	3.4	11.5	11.7	15.8	21.9	28.3	32.3	28.3	22.5	-	-	**6.9**
		Jordan	2008-2012	-	0.1	0.5	0.7	1.2	1.6	2.7	1.9	4.4	8.4	10.5	11.5	14.7	19.3	27.9	17.8	-	-	**4.5**
		Kuwait	2008-2012	-	-	0.4	0.6	0.6	1.3	1.0	1.3	3.6	6.8	12.7	12.0	17.9	29.7	33.7	24.7	24.7	-	**4.8**
		Qatar	2008-2012	-	-	4.1	3.2	-	-	-	-	8.9	17.1	4.2	12.8	50.6	38.1	53.3	60.5	25.7	-	8
		Saudi Arabia, Riyadh	2008-2012	0.2	0.2	0.6	1.2	0.4	1.6	2.0	0.8	3.7	5.5	4.6	7.0	11.1	15.8	27.6	14.5	-	-	**3.3**
		Lebanon	2005-2016	0.06	0.11	0.28	0.84	1.12	1.49	2.18	3.27	8.54	15.47	22.61	25.14	28.37	33.70	29.95	30.82	-	-	**7.9**
	Random	Austria	2008-2012	0.2	0.2	0.9	0.9	1.2	1.5	2.2	4.8	7.5	14.0	19.2	28.6	31.9	35.9	42.0	47.9	53.6	54.2	**8.4**
		Italy, Milan	2008-2012	0.3	1.3	1.8	1.4	2.8	1.7	3.9	5.3	8.9	21.0	26.0	30.8	36.1	37.5	42.0	44.7	45.2	52.7	**10.0**
		USA	2008-2012	0.1	0.3	0.7	1.3	1.6	2.2	3.3	4.8	8.5	14.0	19.3	23.8	30.0	37.2	43.1	47.0	49.2	47.0	**8.4**
		Costa Rica	2008-2011	-	0.3	1.1	1.4	2.3	2.3	2.6	4.8	6.8	9.1	12.0	15.3	15.2	15.2	25.9	18.5	20.6	32.6	**5.3**
		Republic of Korea	2008-2012	0.1	0.4	1.6	2.4	2.5	2.7	3.7	5.5	8.6	12.8	15.5	14.8	15.8	15.8	15.5	16.2	15.5	16.4	**5.8**
		New Zealand	2008-2012	-	-	0.4	2.1	0.7	3.0	3.4	4.7	8.1	12.1	17.5	26.1	28.3	37.9	45.7	41.4	59.6	57.7	**8.3**
